# A HEV-restricted sulfotransferase is expressed in rheumatoid arthritis synovium and is induced by lymphotoxin-α/β and TNF-α in cultured endothelial cells

**DOI:** 10.1186/1471-2172-6-6

**Published:** 2005-03-07

**Authors:** José L Pablos, Begoña Santiago, Durwin Tsay, Mark S Singer, Guillermo Palao, María Galindo, Steven D Rosen

**Affiliations:** 1 Servicio de Reumatología y Unidad de Investigación, Hospital 12 de Octubre, 28041 Madrid, Spain; 2 Department of Anatomy, University of California, San Francisco, California, USA

## Abstract

**Background:**

The recruitment of lymphocytes to secondary lymphoid organs relies on interactions of circulating cells with high endothelial venules (HEV). HEV are exclusive to these organs under physiological conditions, but they can develop in chronically-inflamed tissues. The interaction of L-selectin on lymphocytes with sulfated glycoprotein ligands on HEV results in lymphocyte rolling, which represents the initial step in lymphocyte homing. HEV expression of GlcNAc6ST-2 (also known as HEC-GlcNAc6ST, GST-3, LSST or CHST4), an HEV-restricted sulfotransferase, is essential for the elaboration of L-selectin functional ligands as well as a critical epitope recognized by MECA-79 mAb.

**Results:**

We examined the expression of GlcNAc6ST-2 in relationship to the MECA-79 epitope in rheumatoid arthritis (RA) synovial vessels. Expression of GlcNAc6ST-2 was specific to RA synovial tissues as compared to osteoarthritis synovial tissues and localized to endothelial cells of HEV-like vessels and small flat-walled vessels. Double MECA-79 and GlcNAc6ST-2 staining showed colocalization of the MECA-79 epitope and GlcNAc6ST-2. We further found that both TNF-α and lymphotoxin-αβ induced *GlcNAc6ST-2 *mRNA and protein in cultured human umbilical vein endothelial cells.

**Conclusion:**

These observations demonstrate that GlcNAc6ST-2 is induced in RA vessels and provide potential cytokine pathways for its induction. GlcNAc6ST-2 is a novel marker of activated vessels within RA ectopic lymphoid aggregates. This enzyme represents a potential therapeutic target for RA.

## Background

Rheumatoid arthritis (RA) is an autoimmune disease characterized by chronic inflammation of the synovial membrane that leads to the destruction of cartilage and bone tissues. The major cellular components of the inflammatory infiltrate in RA synovium are T cells, macrophages, B cells, and dendritic cells (DCs) [1]. Tissue-infiltrating lymphocytes are often arranged in follicles which exhibit variable degrees of germinal center (GC) reactions [2-4]. These highly organized structures are similar to secondary lymphoid organs, and the process is referred to as lymphoid neogenesis [5,6]. In RA, this ectopic formation of lymphoid structures correlates with the expression of lymphotoxin-β (LT-β) and homing chemokines such as BLC (CXCL13) and SLC (CCL21), recapitulating the developmental process of lymphoid organogenesis [7].

The recruitment of cellular elements to secondary lymphoid organs relies on interactions between circulating leukocytes and the specialized endothelium of high endothelial venules (HEV), which are exclusive to these organs under physiological conditions. The interaction of L-selectin on lymphocytes with sulfated glycoprotein ligands on HEV of lymph nodes results in lymphocyte rolling on endothelium which represents the initial step in lymphocyte homing [8]. HEV-ligands for L-selectin consist of a set of heavily O-glycosylated glycoproteins, which include GlyCAM-1 and CD34 in the mouse and podocalyxin and CD34 in the human [9]. Recognition of these ligands by L-selectin requires sialylation, fucosylation and sulfation of their mucin-like domains [10]. The minimal recognition epitope appears to be comprised of a capping group known as 6-sulfo sialyl Lewis x (6-sulfo sLex) in which the C-6 position of GlcNAc is esterified with sulfate. The MECA-79 mAb recognises a critical sulfation-dependent determinant on these ligands that overlaps with the L-selectin recognition epitope. Expression of GlcNAc6ST-2 (commonly referred to as HEC-GlcNAc6ST or LSST), an HEV-localized sulfotransferase, is essential for the elaboration of functional ligands within lymph nodes, as well as the generation of the MECA-79 epitope [11-13]. Another sulfotransferase known as GlcNAc6ST-1 also contributes to this epitope in HEV [14]. MECA-79 recognizes a set of sialomucins, including the aforementioned L-selectin ligands, together with another recently identified sialomucin called endomucin [15,16]. The complex is referred to as peripheral node addressin (PNAd) [13]. MECA-79 blocks the attachment of lymphocytes to peripheral lymph node (PN) HEV in vitro and inhibits lymphocyte homing to PN in vivo [17].

The role of GlcNAc6ST-2 in the generation of L-selectin ligands in lymphoid organs has been clearly established in mice genetically deficient in this enzyme [18-20]. Lymphocyte homing to PN is reduced in the *GlcNAc6ST-2 *-/- mice as reflected by a smaller size of the PN and a 60% decrease in the number of total lymphocytes within this organ. In addition to lymphoid organs, induction of the MECA-79 epitope has been identified in several chronically inflamed tissues where it is proposed to participate in leukocyte recruitment [21]. Inflamed synovium contains high-walled vessels, resembling HEV, which express the MECA-79 epitope and are capable of binding L-selectin+ lymphocytes in *ex vivo *assays [21-25].

Recently, the use of GlcNAc6ST-2 specific antibodies has demonstrated de novo induction of this enzyme in HEV-like vessels and its correlation with the presence of the MECA-79 epitope in several mouse models of chronic inflammation [26]. In this study using an antibody to human GlcNAc6ST-2, we analyse whether this enzyme is expressed in RA and in cultured endothelial cells and its correlation with the expression of MECA-79 epitope.

## Results

### Expression of MECA-79 epitope and GlcNAc6ST-2 in synovial tissues

**Figure 1:**
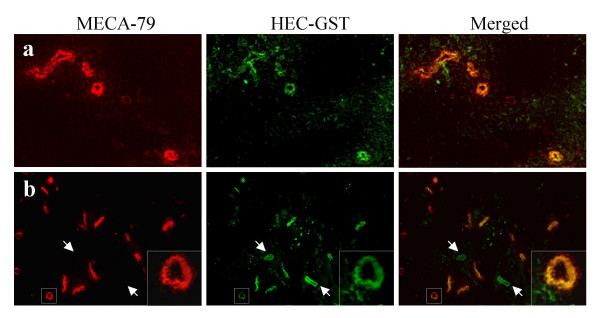
**Double immunofluorescent detection of the MECA-79 epitope and GlcNAc6ST-2 in human lymph node and RA synovial tissues. **Sections were simultaneously labelled with MECA-79 (red) and anti-GlcNAc6ST-2 (green) antibodies and photographed under appropriate filters. On the right panels, a merged image shows colocalization of both markers (yellow). (a) Lymph node. (b) RA synovium; Two MECA-79 negative and GlcNAc6ST-2 positive vessels are marked by arrows. Original magnification (a) 200×. (b) 150×. Selected areas within squared insets are shown on the lower right corner insets with higher magnification (630×).

We produced an anti-human GlcNAc6ST-2 antibody and tested it on human lymph node and tonsil sections. A vascular pattern of labelling was observed which colocalized to MECA-79 positive vessels of HEV morphology (Figure 1a). A few mononuclear cells were also labelled with the anti GlcNAc6ST-2 antibody. Expression of the MECA-79 epitope was detected in 7 of 14 RA and none of the 9 OA synovial tissues. Most MECA-79^+ ^vessels showed a HEV morphology but in some cases, small vessels with a flat endothelium were also labelled. Five of the 7 MECA-79^+ ^tissues contained abundant lymphocytic infiltrates, which in 4 cases displayed a follicular pattern, and in 2 cases, MECA-79^+ ^vessels were observed in the absence of significant infiltration (Table 1). Three MECA-79 negative RA tissues contained diffuse lymphocytic infiltrates and in one case, a single follicular infiltrate.

**Figure 2:**
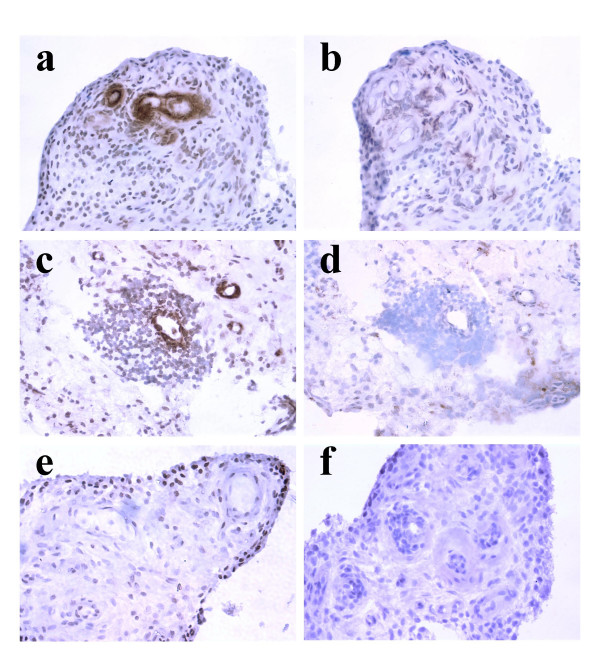
**Immunoperoxidase staining of RA and OA synovial tissue with anti-GlcNAc6ST-2 antibodies. **RA (a-d) or OA (e, f) synovial tissue sections were immunostained with anti-human GlcNAc6ST-2 (a, b, e) or anti-mouse GlcNAc6ST-2 (c, d, f) antibody. Color was developed with DAB peroxidase substrate (brown color) and sections were counterstained with hematoxylin. Sections b and d are serial to a and c respectively, and were preincubated with human or mouse GlcNAc6ST-2 peptide to demonstrate the specificity of immunostaining. Original magnification: 400×.

Expression of GlcNAc6ST-2 was not detected in any of the OA synovial tissues. In contrast, 10 of 14 RA tissues showed GlcNAc6ST-2 by immunoperoxidase staining (Figure 2). Staining was located to endothelial cells of HEV vessels in the sublining and in some samples, also in small vessels with flat endothelium. In some sections, a few perivascular mononuclear cells were also stained. Double MECA-79 and GlcNAc6ST-2 immunofluorescence showed that all MECA-79 positive vessels also displayed GlcNAc6ST-2 (Figure 1b). However, in a low proportion of GlcNAc6ST-2^+ ^vessels, MECA-79 was not detected.

Specificity of the immunoperoxidase GlcNAc6ST-2 labelling was confirmed by substituting normal rabbit IgG for the specific antibody. There was no detectable staining (data not shown). Preincubation of the anti-GlcNAc6ST-2 antibody with the peptide immunogen abrogated the staining (Figure 2b). We also immunostained with a polyclonal antibody raised against murine GlcNAc6ST-2 [26] and observed a similar pattern of staining in small vessels (Figure 2c). This antibody reacted with smooth muscle cells in the media of larger vessels which we attribute to a spurious cross-reactivity (data not shown). Immunostaining with this antibody was also abrogated by preincubation with murine GlcNAc6ST-2 peptide (Figure 2d).

### Expression of GlcNAc6ST-2 by cultured HUVEC

To analyze the potential role of LT-αβ and TNF-α, two cytokines abundantly expressed in RA synovium and associated with lymphoid neogenesis and GlcNAc6ST-2 expression in transgenic models [3-7,26,28], we examined the effects of the exposure to these cytokines on GlcNAc6ST-2 expression in human endothelial cells lacking the HEC phenotype (HUVEC).

**Figure 3:**
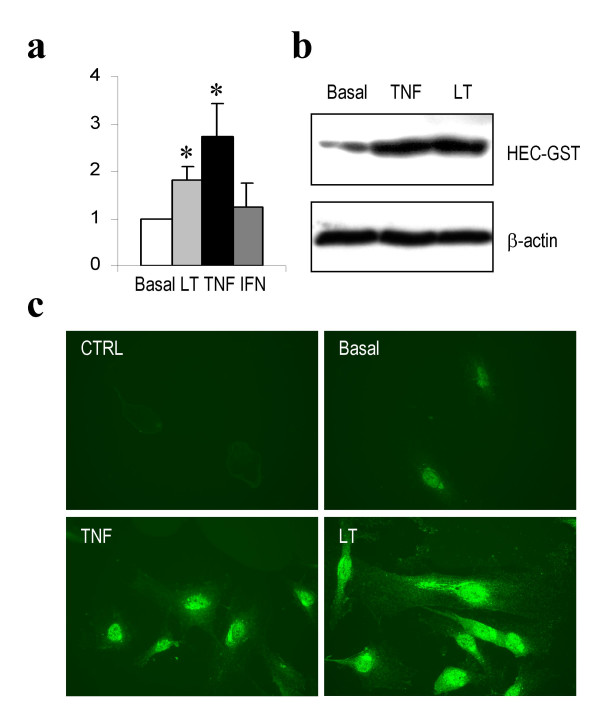
**Expression of GlcNAc6ST-2 in cultured HUVEC. **(a) Real time RT-PCR quantification of *GlcNAc6ST-2 *mRNA in cultured HUVEC. HUVEC cultures were stimulated for 8 h with LT-αβ (LT), TNF-α (TNF), or interferon-γ (IFN) and mRNA obtained from stimulated and non-stimulated cells. Basal (non-stimulated) expression was set to 1 and the *GlcNAc6ST-2/β-actin *ratio in stimulated cells is shown (mean ± SD of three independent experiments, each including triplicate HUVEC cultures). (*) p < 0.05. (b) Western blot analysis of GlcNAc6ST-2 in cultured HUVEC stimulated for 24 h with LT-αβ (LT) or TNF-α (TNF), (data are representative of three independent experiments with different HUVEC lines).(c) Immunofluorescent detection of GlcNAc6ST-2 protein in cultured HUVEC endothelial cells non-stimulated (Basal) or stimulated for 24 h with LT-αβ (LT) or TNF-α (TNF). In control (CTRL) panel, anti-GlcNAc6ST-2 antibody was replaced by non-immune rabbit IgG (images are representative of three independent experiments).

By RT-PCR, treatment with LT-αβ or TNF-α for 8 h induced a significant increase in the expression of *GlcNAc6ST-2 *mRNA in HUVEC (Figure 3a). A synergistic effect of treatment with both cytokines was not detected (data not shown). Treatment with an unrelated cytokine (interferon-γ) did not significantly modify *GlcNAc6ST-2 *mRNA expression levels.

By western blot analysis with the anti-human GlcNAc6ST-2 antibody, we detected a single 50 kD band in HUVEC protein extracts, consistent with the expected MW for human GlcNAc6ST-2 [11,12]. GlcNAc6ST-2 protein expression was significantly increased after 24 h of TNF-α or LT-αβ treatment in HUVEC (Figure 3b). Immunofluorescent labelling of HUVEC cultures with anti-human GlcNAc6ST-2 antibody showed a weak, but clearly detectable signal above the non-immune rabbit IgG control. Pretreatment with either TNF-α or LT-αβ induced a clear increase of immunofluorescence with a perinuclear distribution which is characteristic of the Golgi apparatus (Figure 3c). Neither TNF-α nor LT-αβ treatment induced expression of the MECA-79 epitope in HUVEC (data not shown).

## Discussion

The presence of MECA-79^+ ^vessels has been previously reported in human inflammatory lesions, often associated with the development of lymphoid neogenesis [21,26]. In a variety of unrelated inflammatory conditions (rejecting heart and kidney allografts, peribronchial specimens from asthmatics, thyroiditis, psoriasis, and *H. pylori *infection) induction of MECA-79^+ ^vessels in association with inflammatory infiltrates has been demonstrated [29,32]. In some cases, MECA-79^+ ^vessels also display HEV-like morphology although flat endothelium of inflammatory vessels can also express the MECA-79 epitope. In chronic arthritis synovial tissues, the presence of HEV and MECA-79^+ ^vessels has been previously demonstrated [22,23]. Recently, the pathophysiological significance of MECA-79^+ ^vessels has been directly demonstrated in a sheep model of asthma. Pretreatment of asthmatic sheep with intravenously administered MECA-79 blunted both late-phase airway responses and airway hyperresponsiveness induced by airway allergen challenge [33].

In murine models of lymphoid neogenesis, including spontaneous models (AKR mouse and NOD mouse), and transgenic models (RIP-BLC and RIP-LT-αβ), expression of the MECA-79 epitope is closely associated with the induction of GlcNAc6ST-2 [12,26,28]. Taking advantage of a newly-generated antibody to human GlcNAc6ST-2, our present results are the first to show this association in a chronic inflammatory disease in humans. Several of our observations suggest that the induction of GlcNAc6ST-2 represents an early event in the process of lymphoid neogenesis within RA synovium. In the first place, GlcNAc6ST-2 was expressed in flat and high endothelial vessels which exceeded the number of MECA-79^+ ^vessels in double-labelled sections. Secondly, GlcNAc6ST-2 and MECA-79 reactivity were detected in some vessels which were not associated with lymphoid aggregates, and in a few cases, even in the absence of significant mononuclear cell infiltration. The latter observation also suggests that MECA-79^+ ^vessels may contribute to lymphoid recruitment, but additional factors are necessary. This is consistent with the multistep model of leukocyte transendothelial migration to lymphoid organs, where posting of chemokines on endothelium and integrin-mediated adhesion of rolling leukocytes are required [8]. Lack of expression of MECA-79 epitope and GlcNAc6ST-2 in OA samples suggests that the scanty infiltration of OA tissues is not sufficient to induce these features and that RA-associated factors are needed.

The development of lymphoid neogenesis in RA synovium has been suggested to facilitate local autoimmune responses permitting the development of B cells and local production of autoantibodies and immune complex formation [34-36]. The recent development of B-cell depleting therapy demonstrates an important role for these cells in RA [37]]. The recruitment of L-selectin^+ ^dendritic cells, B-cells and CD45RA T-cells to RA synovium correlates with the expression of homing chemokines such as CXCL13, CCL19 and CCL21, and the latter partially colocalizes to MECA-79 positive HEV [3,38,39]. We have also detected expression of CXCL12, a homing chemokine abundantly present in RA tissues in HEV-like vessels [40].

Local expression of LT-β and TNF-α are important factors in the pathogenesis of RA, and LT-β expression correlates with the expression of homing chemokines and lymphoid neogenesis [1,3]. The role of LT-αβ and homing chemokines in the induction of HEV has been suggested by murine studies in RIP-LT-αβ and RIP-BLC (CXCL13) mice which spontaneously develop lymphoid aggregates and GlcNAc6ST-2 expression in MECA-79^+ ^vessels [26,28,29]. The role of this enzyme in lymphoid recruitment has also been studied in RIP-BLC/ *GlcNAc6ST-2*-/- mice which exhibit a reduction in lymphoid infiltration [26].

We demonstrate that de novo induction of GlcNAc6ST-2 expression also occurs upon LT-αβ or TNF-α stimulation in cultured HUVEC, further supporting the role of LT-αβ and TNF-α as major inducers of extranodal lymphogenesis. Enforced LT-αβ expression in murine models of lymphoid neogenesis induces the expression of homing chemokines, making it difficult to dissect the role of LT-αβ and chemokines in GlcNAc6ST-2 expression and HEV-development [41]. A role for LT-αβ, which is downstream of chemokines, has been observed in a transgenic model of BLC expression by genetic or functional targeting of LT-αβ [42]. In murine models of extranodal lymphoid neogenesis, a non redundant role for LT-αβ signaling through the alternative NF-κB pathway has been demonstrated, whereas the role for TNF-α seems dispensable and ectopic LT-α does not induce GlcNAc6ST-2 expression (13, 28). Our data suggest that in human cultured EC TNF-α may contribute to this process by inducing GlcNAc6ST-2 although its relevance *in vivo *remains to be determined. Therefore, our data suggest that both LT-αβ and TNF-α can directly induce GlcNAc6ST-2 in cultured endothelial cells, although additional factors seem to be required for the full development of the HEC phenotype *in vitro*. One such factor may be the core 1 extension enzyme, known as Core1-β 3GlcNAcT, which is required for the formation of the MECA-79 epitope on O-glycans [29,43]. Our results further establish carbohydrate modifying enzymes as an important group of transcriptional targets for cytokines during inflammation. Increased carbohydrate sulfation of several glycoproteins, including heparan sulfate proteoglycans, CD44 and airway mucins, has previously been demonstrated in response to TNF-α [44-46].

In RA, TNF-α plays a major and multifunctional role in the pathogenesis of the disease whereas the role of LT-αβ is currently being explored [47]. The potential contribution of these factors to the development of HEV expands the spectrum of possible pathogenetic roles for these cytokines.

## Conclusion

MECA-79 defined glycoproteins serve as ligands for L-selectin during the process of lymphocyte homing to lymph nodes. This class of ligands is also critical for inflammatory leukocyte trafficking and the associated disease, as recently demonstrated in a sheep model of asthma. Previous experiments in the mouse have shown the requirement for GlcNAc6ST-2 in generating the MECA-79 epitope and the associated L-selectin ligand activity in HEV and HEV-like vessels. Here, we demonstrate that MECA-79^+ ^vessels within human RA synovium also express GlcNAc6ST-2, strongly implying that this enzyme contributes to the ligands on these vessels. Establishing the direct participation of GlcNAc6ST-2 in the formation of L-selectin ligands on RA HEV would identify this enzyme as a potential therapeutic target for inhibiting leukocyte recruitment to the joints. In the same context, our finding that TNF-α induces GlcNAc6ST-2 in cultured endothelial cells may help to rationalize the efficacy of anti TNF-α therapy in the treatment of RA and other inflammatory diseases. Moreover, the activity of LT-αβ in inducing GlcNAc6ST-2 in HUVEC is likely related to the established requirement for this cytokine in the formation of HEV during lymphoid organ development. Many opportunities for future investigation are clearly indicated.

## Methods

### Preparation of anti-human GlcNAc6ST-2 antibody

A polyclonal antibody against human GlcNAc6ST-2 was prepared following the same basic procedures used for mouse GlcNAc6ST-2 [26]. The peptide chosen, RGKGMGDHAFHTNC, differed from that of the corresponding mouse sequence by one amino acid (D replacing Q) with the C-terminal C added to facilitate conjugation. The peptide was coupled to KLH and injected into New Zealand White rabbits by Covance Research Products (Denver, PA). 500 μg of conjugated peptide was mixed with 0.5 ml complete Freund's adjuvant and injected intradermally, followed by three boosts, every three weeks, with subcutaneous injections of 250 μg conjugated peptide in 0.5 ml incomplete Freund's adjuvant. The subsequent antiserum was subjected to purification on the immunogen peptide coupled to agarose using the SulfoLink Kit (Pierce Biotechnology, Rockford, IL), following the manufacturer's recommendations. The affinity purified antibody was washed into Dulbecco's PBS on a Centricon 30 (Millipore, Billerica, MA) and concentrated to 0.125 mg/ml.

### GlcNAc6ST-2 and MECA-79 immunolabeling

Synovial tissues were obtained from 14 RA and 9 control (OA) patients at the time of knee prosthetic replacement surgery. All patients gave informed consent, and the study was approved by the ethics committee of the Hospital 12 de Octubre.

Tissues were snap frozen in OCT and stored at -80°C. Human tonsil and lymph nodes frozen sections were used as controls for MECA-79 and GlcNAc6ST-2 immunolabeling.

Frozen sections (8 μm) were fixed for 10 minutes in cold acetone. Double GlcNAc6ST-2 and MECA-79 immunolabeling was performed by blocking for 30 minutes in 5% normal goat serum PBS, followed by incubation of sections with 1.4 μg/ml MECA-79 mAb (rat IgM) and 0.3 μg/ml rabbit anti-human GlcNAc6ST-2 at room temperature for 1 hour. After washing in PBS, a secondary biotinylated goat anti-rabbit IgG at 1.3 μg/ml was added (Jackson Immunoresearch Laboratories, West Grove, PA). Fluorescence was developed with 1.5 μg/ml Cy3-conjugated goat anti-rat IgM and 1.8 μg/ml Cy2-conjugated streptavidin (Jackson Immunoresearch Laboratories).

Immunoperoxidase staining was performed by overnight incubation at 4°C with either anti-human or anti-mouse GlcNAc6ST-2. Endogenous peroxidase was blocked in 0.3 % H_2_O_2 _in methanol. Immunoperoxidase detection was performed by ABC method according to the manufacturer (Vectastain^®^, Vector Laboratories, Burlingame, CA). Peroxidase activity was developed by diaminobenzidine substrate and slides were counterstained in Gills hematoxylin. Controls with non-immune rabbit IgG at the same concentration as anti-human or anti-mouse GlcNAc6ST-2 antibodies were included. Additional controls were performed by preincubating sections with human or mouse GlcNAc6ST-2 peptide at 10 μg/ml. To ensure identical conditions, RA and OA sections were mounted on the same slide and all slides were processed in parallel.

### GlcNAc6ST-2 expression in HUVEC cultures

Human umbilical vein endothelial cells (HUVEC) were prepared from umbilical cords by collagenase digestion and were propagated in medium 199 (Life Technologies, Paisley, Scotland) with 20% FCS. Cultured cells were characterized as EC by flow cytometry with anti-endothelial cell antibody P1H12 (Chemicon International, Temecula, CA). The presence of HEC in these cultures was excluded by flow cytometry with MECA-79 mAb.

HUVEC cultures were stimulated with either 25 ng/ml of TNF-α, 20 ng/ml of LT-α1/β2, or 200 ng/ml of interferon-γ (R&D Systems, Inc., Abingdon, UK) for 8–24 h and total RNA or protein were extracted. For immunofluorescent detection of GlcNAc6ST-2, HUVEC were cultured on glass coverslips and similarly treated. Coverslips were fixed with 4% paraformaldehyde and immunofluorescent detection of GlcNAc6ST-2 was performed as described above for tissue sections.

RT-PCR was performed on cDNA synthesized from 1 μg of total RNA of EC cultures using the following *GlcNAc6ST-2 *oligonucleotides: upstream 5'-GCAGCATGAGCA AAAACTCAAG-3' and downstream 5'-TCCAGGTAGACAGAAGATCCAG-3', and β-actin oligonucleotides upstream 5'-CTACCTCATGAAGATCCTCAC-3' and downstream 5'-GTCCACGTCACACTTCATGATG-3'. Real-time monitoring of PCR reactions was performed on a Roche LightCycler instrument, using SYBR Green I kit (Roche Diagnostics, Mannheim, Germany). The specificity of the amplicons was checked by electrophoresis which demonstrated single transcripts of 449 bp (*GlcNAc6ST-2*) and 303 bp (*β-actin*) as expected. For relative quantification, we calculated the n-fold differential expression by the ΔC_t _method (Ct denoting the threshold cycle of PCR amplification at which product is first detected by fluorescence) that compares the amount of target gene amplification, normalized to the β-actin endogenous reference as previously described [27].

Western blot detection of GlcNAc6ST-2 was performed on HUVEC protein extracts (50 μg) electrophoresed on a 10% polyacrylamide gel and electrophoretically transferred to nitrocellulose filters. Membranes were incubated overnight with either rabbit anti-human GlcNAc6ST-2 or control anti-β actin (clone AC-15, Sigma-Aldrich Química, Spain) antibodies in 5% non-fat dried milk-TBST. The filters were washed and incubated for 1 h with secondary antibodies linked to peroxidase at 0.08 μg/ml (Santa Cruz Biotechnology, Santa Cruz, CA). Bands were visualized by an enhanced chemiluminiscence system (Pierce, Rockford, IL).

## Authors' contributions

JLP carried out the immunodetection studies and participated in the study design and coordination. BS performed the studies in cultured endothelial cells. MG and GP participated in the selection of patients, and the collection and preparation of synovial tissues. DT and MSS prepared the antibodies and developed the immunodetection techniques. SDR participated in the design and coordination of the study. All authors contributed to drafting the manuscript and have read and approved the final version.

## Figures and Tables

**Table 1:** Summary of immmunodetection of the MECA-79 epitope and GlcNAc6ST-2 in RA tissues.

Tissues	Lymphocytic infiltration	MECA-79	GlcNAc6ST-2
RA1	Follicular	+	+
RA2	Follicular	+	+
RA3	Follicular	+	+
RA4	Follicular	+	+
RA5	Diffuse	+	+
RA6	Negative	+	+
RA7	Negative	+	+
RA8	Follicular	-	+
RA9	Diffuse	-	+
RA10	Negative	-	+
RA11	Diffuse	-	-
RA12	Negative	-	-
RA13	Negative	-	-
RA14	Negative	-	-
